# Pathological mechanisms of connexin26-related hearing loss: Potassium recycling, ATP-calcium signaling, or energy supply?

**DOI:** 10.3389/fnmol.2022.976388

**Published:** 2022-09-15

**Authors:** Penghui Chen, Wenjin Wu, Jifang Zhang, Junmin Chen, Yue Li, Lianhua Sun, Shule Hou, Jun Yang

**Affiliations:** ^1^Department of Otorhinolaryngology-Head and Neck Surgery, Xinhua Hospital, Shanghai Jiao Tong University School of Medicine, Shanghai, China; ^2^Ear Institute, Shanghai Jiaotong University School of Medicine, Shanghai, China; ^3^Shanghai Key Laboratory of Translational Medicine on Ear and Nose Diseases, Shanghai, China

**Keywords:** connexin, cochlea, gap junction, hearing loss, mechanism

## Abstract

Hereditary deafness is one of the most common human birth defects. *GJB2* gene mutation is the most genetic etiology. Gap junction protein 26 (connexin26, Cx26) encoded by the *GJB2* gene, which is responsible for intercellular substance transfer and signal communication, plays a critical role in hearing acquisition and maintenance. The auditory character of different Connexin26 transgenic mice models can be classified into two types: profound congenital deafness and late-onset progressive hearing loss. Recent studies demonstrated that there are pathological changes including endocochlear potential reduction, active cochlear amplification impairment, cochlear developmental disorders, and so on, in connexin26 deficiency mice. Here, this review summarizes three main hypotheses to explain pathological mechanisms of connexin26-related hearing loss: potassium recycling disruption, adenosine-triphosphate-calcium signaling propagation disruption, and energy supply dysfunction. Elucidating pathological mechanisms underlying connexin26-related hearing loss can help develop new protective and therapeutic strategies for this common deafness. It is worthy of further study on the detailed cellular and molecular upstream mechanisms to modify connexin (channel) function.

## Introduction

Up to now, 21 human genes and 20 mouse genes encoding connexin (Cx) have been identified, of which 19 are considered homologous pairs (Söhl and Willecke, 2003). All Cxs are considered to share the same topology, with cytoplasmic amino and carboxyl terminals, and four transmembrane domains are connected by two extracellular rings and one cytoplasmic ring (Beyer and Berthoud, 2018). In the cochlea, there are varieties of Cxs including Cx26, Cx29, Cx30, Cx31, Cx32, Cx43, and Cx45, which are named according to their molecular weight size (Lautermann et al., 1998; Ahmad et al., 2003; Buniello et al., 2004). Different connexins have distinct distribution and expression characteristics during the development of cochlea (Forge, 1984). Cx26 and Cx30 are the prevailing Cxs in the developing and mature rodent cochlea (Tsutsui et al., 1976; Kikuchi et al., 1995; Zhao and Yu, 2006). Six connexins can combine into one single junctional hemichannel, and two hemichannels between two adjacent cells form a gap junction (GJ) (Sáez and Leybaert, 2014). The GJ which is composed of the same connexin protein is known as homomeric gap junction, while the GJ which is composed of heteromeric connexin is also known as heterotrimeric gap junction (Kumar and Gilula, 1996). GJ channels and hemichannels generally allow the passage of ions (K+), some second messengers [adenosine-triphosphate (ATP) and inositol-1,4,5-trisphosphate (IP3)],(Niessen et al., 2000; Beltramello et al., 2005; Bedner et al., 2006; Hernandez et al., 2007) and metabolites (glycolytic intermediates, vitamins, amino acids, and nucleotides) (Zou et al., 2005; Chang et al., 2008; Kanaporis et al., 2008) of molecular weight less than 1.4 kDa molecular weight or diameter less than 1.5 nm (Leybaert et al., 2017). The hemichannel is mainly maintained in a closed state, and its opening and closing are mainly regulated by (1) extracellular Ca^2+^ and Mg^2+^ concentrations (Verselis and Srinivas, 2008; Bennett et al., 2016), (2) membrane potential (Verselis et al., 1994), and (3) post-translational modifications of proteins (e.g., phosphorylation) (Moreno, 2005; Aasen et al., 2018).

Mammalian cochlear inter-cellular connections are divided into two main cellular network systems, namely the epithelial gap junction system (E-sys) and the connective tissue gap junction system (C-sys) (Kikuchi et al., 1995). In mammals, the E-sys forms around embryo day 19 (E19) and is well developed by postnatal day5 (P5). E-sys is located in supporting cells around the organ of Corti, the bordering epithelial cells of the inner sulcus and outer sulcus, the interdental cells of the spiral limbus, and the root cells (Jagger and Forge, 2013) that extend their process into the spiral ligament. C-sys develops around P0 and is further divided into two systems, (1) fibrocytes of the spiral limbus; (2) fibroblasts, basal cells, and intermediate cells of the *stria vascularis* (SVs), fibroblasts of the suprastrial region, mesenchymal cells of the vestibular scala, and dark cells (Kikuchi et al., 2000).

The maintenance of normal function of the inner ear depends on the homeostasis of three fluid environments—the perilymph fluid (cerebrospinal fluid), the endolymphatic fluid, and the intracellular fluid. Furthermore, the maintenance of the fluid environment homeostasis depends on the cellular network system of substance exchange and signaling transmission which is formed by intercellular GJ channels and extracellular hemichannels.

Gap junction protein 26 (connexin26, Cx26) encodes by the *GJB2* gene. *GJB2* mutations cause about 50% of non-syndromic hearing loss. There are more than 340 mutations of *GJB2* (Mammano, 2019), including missense mutation, nonsense mutation, frameshift mutation, insertion mutation, deletion mutation, and so on. Most *GJB2* mutations cause recessive non-syndromic deafness (DFNB1A, OMIM: 220290). *GJB2* mutations affect the following: (1) the protein expression level (Thönnissen et al., 2002); (2) their transport to the plasma membrane; (3) their channel biological characteristics (voltage gated, chemical gated, and channel permeability) (Gerido et al., 2007).

Cx26 is the most common and harmful deafness gene. Cx26 is responsible for intercellular substance transfer and signal communication and plays a critical role in hearing acquisition and maintenance. Cx26 mutations can not only cause congenital deafness but also cause delayed deafness. The deafness mechanism caused by Cx26 mutation is not clear. Mouse models are widely used in hearing and deafness mechanism research (Leibovici et al., 2008). Benefit from the development of transgenic technologies such as the Cre-loxP system and the establishment of the Cx26 conditional knockout mouse model has promoted the study of the mechanism of Cx26 mutation deafness (Gridley and Murray, 2022). This paper reviews the research progress of congenital deafness and delayed deafness caused by Cx26 mutation in recent years and tries to find the underlying pathological mechanisms of connexin26-related hearing loss.

## Mouse models of connexin 26 deficiency

Given the complex phenotype and mutation in Cx26-related hearing loss, it is difficult to explore the underlying pathogenesis mechanism. More and more Cx26 transgenic mice have been used to study pathogenesis mechanisms. We summarized and classified Cx26 transgenic mice into two major types based on the deafness phenotype: profound congenital deafness model mice and late-onset progressive deafness model mice. Profound congenital deafness model mice include Gjb2^loxP/loxP^; Otog-Cre, Gjb2^loxP/loxP^; Sox10-Cre, Gjb2^loxP/loxP^; Pax2-Cre, Gjb2^loxP/loxP^; Foxg1-Cre, Gjb2^loxP/loxP^; Rosa26-Cre, Gjb2^loxP/loxP^; Prox1-CreERT2, and Gjb2^loxP/loxP^; Rosa26cre-ER injected with tamoxifen at E19 or P1 (Sun et al., 2009; Wang et al., 2009; Chang et al., 2015). The common pathological changes of these mice are the failure of the opening of the tunnel of Corti at P6, serious hair cell loss from the middle turn after P14, and the secondary loss of spiral ganglion neurons (Cohen-Salmon et al., 2002; Sun et al., 2009; Wang et al., 2009). Obviously, in Cx26 deficiency mice, the sensory epithelial cell injury precedes hearing loss. The failure of the tunnel of Corti to open is a landmark event (Lin et al., 2013). Since the tunnel of Corti and Nuel’s space are not developed, perilymph failed to infiltrate around the outer hair cell body, resulting in an effective K^+^ potential difference (endocochlear potential, EP), and cochlear amplifier function fail to form (Wang et al., 2009). Researchers found that the reduction of microtubules in inner and outer column cells is likely the reason that the tunnel of Corti failed to open (Lin et al., 2013; Xie et al., 2019). Cx26 plays a crucial role in the early development of the cochlea. The developmental disorder of supporting cells may be the main mechanism of congenital profound deafness caused by Cx26 deficiency.

Cx26 model mice that present late-onset progressive deafness mainly include p.V37I homozygous mutant mice, Cx26^±^ mice, and Gjb2^loxP/loxP^; Rosa26cre ER mice which received injection with tamoxifen at P5, P8, and later (Zhu et al., 2015; Chen et al., 2016; Lin et al., 2019). All these mice acquire normal hearing function and show normal cochlear development without hair cell loss at P30 (Chang et al., 2015). However, with aging, progressive hearing loss first started only at high frequency and gradually extended to full frequencies (Chang et al., 2015; Xie et al., 2019). It is like the phenotype of DFNA3 or DFNB1. The common pathophysiological alterations of these model mice are the active cochlear amplification impairment which showed that distortion product otoacoustic emission (DPOAE) failed to evoke at an early stage (Chen et al., 2021). With aging, hair cells at the basal turn first start to damage and then gradually expand to the middle and apic turns (Fetoni et al., 2018). This pathological change pattern is like the pattern of noise-induced deafness and age-related deafness (Zhou et al., 2016). This kind of mice model further proves that Cx26 plays an essential role in maintaining hearing function, especially in maintaining the active amplification of the cochlea. The rest of the cochlear blood supply depends on two vascular networks, one serving the spiral limbus, and another serving the spiral ligaments and SVs (Figure 2).

## Pathological mechanisms of connexin26-related hearing loss

### Cx26 mutation leads to disruption of potassium recycling in cochlear lymph

In 1983, Santos-Sacchi and Dallos proposed the hypothesis of the GJ function in potassium recycling (Santos-Sacchi and Dallos, 1983). The cochlear GJ is a specific potassium ion channel. By GJ, expelled potassium ions from hair cells are sunken into supporting cells and are eventually transported back to the endolymph.

**FIGURE 1:**
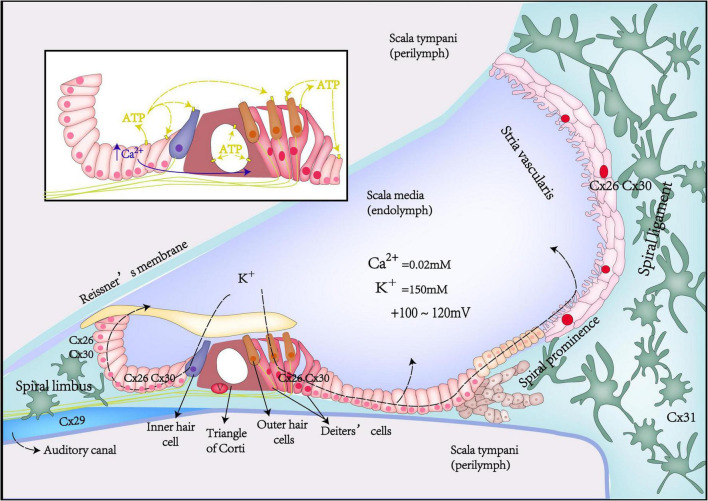
Schematic diagrams of Cx expression, potassium recycling, and ATP-Ca^2+^ signaling in the inner ear.

Potassium recycling is such a process that potassium flows through ion channels in the cochlear GJ system from the perilymph into the endolymph, participating in the formation of hair cell receptor potentials and stable endocochlear potential (EP), finally returning to the perilymph (Lv et al., 2021; Figure 1). Potassium recycling is thought to be critical for maintaining high endolymphatic potassium concentrations and EP (Chen et al., 2021).

**FIGURE 2:**
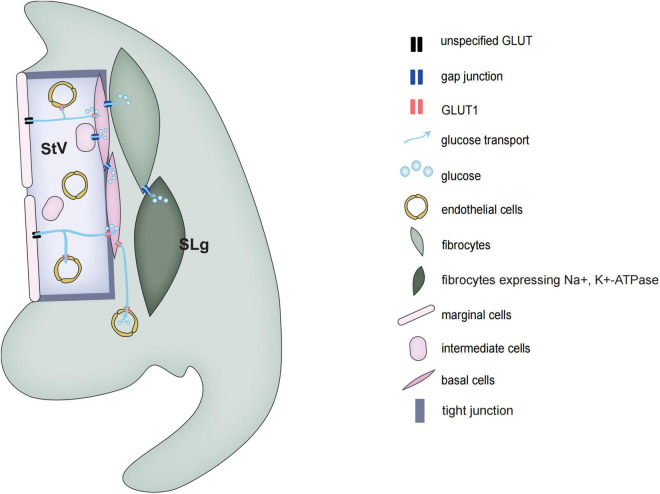
Schematic diagrams of glucose transport in the stria vascularis and spiral ligament of the cochlea.

The cochlea is filled with endolymph and perilymph of unique and different ionic environments. The ionic composition of endolymph is different from that of perilymph. Such as, the potassium concentration in the endolymph is 30 times higher than that of the perilymph, in contrast, Na^+^ concentration in the perilymph is 10 times higher than that in the endolymph. This leads to the potential difference between different parts of the cochlea in the resting state. EP is the positive voltage (+80 mV) of cochlear endolymph in scale media at the resting state. Once acoustic stimulation, the positively charged EP becomes the driving force to promote potassium ions of endolymph in the scala media to pass through the mechano-transduction channel in the top of hair cells and generates auditory receptor current and potential. Thus, the positive EP is necessary for hearing maintenance (Chen and Zhao, 2014; Chen et al., 2021). Gap junction coupling is required to produce positive EP. Loss of Cx26 expression in the cochlea can lead to decreased EP (Chen et al., 2014). Compared with WT mice, Cx26 KO mice had about a 50% reduction in EP (Cohen-Salmon et al., 2002).

The EP reduction attenuates the potassium inflow driving force into hair cell stereocilia and weakens outer hair cell (OHC) electromotility. OHC electromotility serves as a major source of active cochlear amplification in mammals (Ashmore, 2008; Liu et al., 2019). Active cochlear amplification can increase hearing sensitivity and frequency selectivity (Dallos, 2008; Zheng et al., 2021). Therefore, Cx26 deficiency in the cochlear supporting cells can impair active cochlear amplification with DPOAE reduction (Lin et al., 2019).

Based on GJ functions in the cochlea, researchers have hypothesized that the pathological mechanism of deafness caused by Cx26 mutations may be due to dysfunction of GJ, leading to impair potassium recycling in the cochlea. They hypothesized that *GJB2* mutations produce functionally defective or non-functional Cx26 proteins that affect the permeability of the cochlear GJ, impairing GJ coupling and disrupting potassium recycling, and also (1) leading to potassium excessive accumulation in extracellular space near hair cell, generating cell toxicity and eventually damaging the hair cells (Salt and Ohyama, 1993; Teubner et al., 2003; Wangemann, 2006; Zhao, 2017); (2) leading to EP reduction; and (3) leading to impair active cochlear amplification (Kamiya et al., 2014; Chang et al., 2015).

The potassium recycling dysfunction hypothesis can explain the pathogenesis of most of the GJ-related hearing loss including profound congenital hearing loss and late-onset progressive hearing loss. However, there has been no direct evidence found to support this hypothesis so far. Moreover, more and more model mice have been studied, and the theory of potassium recycling has been challenged and questioned in many ways (Zhu et al., 2015). For example, many mutations do not affect the ion permeability of the GJ but still cause deafness (Beltramello et al., 2005); R75W mutant mice exhibit severe deafness but have normal EP (Inoshita et al., 2008), and so on. Therefore, in our opinion, the Cx26 mutation causing impaired potassium recycling may not be its main pathogenic mechanism. The hypothesis of potassium recycling defect cannot explain that Cx26 deficiency can lead to congenital deafness and delayed deafness. The congenital deafness caused by Cx26 mutation is not due to the degeneration of cells and the reduction of cochlear potential but may be due to the developmental disorder of the cochlea itself.

## Critical role of ATP triggered intercellular Ca^2+^ signaling in cochlear development

As an intercellular channel, GJ also plays an important role in intercellular Ca^2+^ signaling transduction (Sirko et al., 2019). Calcium signaling is involved in a variety of cell pathophysiological processes, which is not only the main driving force of cell proliferation and growth but also closely related to cell death (Sirko et al., 2019).

The spread of intercellular Ca^2+^ waves can be realized through transmitting second messengers (Ca^2+^ and IP_3_ by GJ channels, or ATP and IP_3_ by hemichannels). During cochlear development, supporting cells of the Kölliker’s organ can spontaneously and rhythmically release ATP to the endolymphatic surface *via* hemichannels, as well as to adjacent supporting cells *via* GJ channels (Mese et al., 2011; Sellitto et al., 2021). Then, ATP can activate the G protein-coupled P2 purinergic receptor (P2R) of the adjacent cells, producing phospholipase C (PLC)-dependent IP_3_, which activates the endoplasmic reticulum IP_3_ receptor and promotes endoplasmic reticulum calcium release, thereby inducing Ca^2+^ signaling (Piazza et al., 2007). Initially, intracellular Ca^2+^ release from supporting cells usually starts from a small group of cells (2–4) and then passes rapidly through gap junction channels, synchronizing the entire Kölliker’so rgan syncytium, causing cumulative ATP release, and eventually activating the P2Rs on adjacent inner hair cells (IHC), which again leads to causing depolarization of IHCs and the release of calcium-dependent glutamate from ribbon synapses, activating spiral ganglion neurons (SGNs) to generate action potentials(Ceriani et al., 2016). This process is called as the ATP-triggered intercellular Ca^2+^ signaling pathway or sound-independent spontaneous electrical activity, which is a key transient physiological activity during auditory development (Ceriani et al., 2016). With the disappearance of Kölliker’s organ, mature synapses of hair cells start to be established for chemical-electrical connection with postsynaptic afferent nerves, and the abovementioned transient physiological activity ends. Thus, ATP triggered intercellular Ca^2+^ signaling pathway plays a critical role in promoting the maturation of hair cells and SGNs and the refinement of synapses and nerve fibers.

In addition, the propagation of the intercellular Ca^2+^ wave activates and opens TMEM16A (a Ca^2+^-activated chloride channel), causing osmotic cell contraction and wrinkle movement of tall columnar cells of Kölliker’s organ (Tritsch et al., 2007). TMEM16A is highly expressed in columnar supporting cells near IHC. The wrinkle movement of columnar cells will also depolarize IHC and increase the frequency of spontaneous Ca^2+^ action potentials (APs) of IHC at the prehearing stage, thus triggering synaptic vesicle exocytosis and promoting the development and maturation of IHC and SGN at prehearing stage (Wang et al., 2015).

Connexin, as the core of ATP-triggered intercellular Ca^2+^ signaling pathway, and its defect will lead to the disruption of calcium signal transmission. Both Gjb2^loxP/loxP^; Sox10-Cre mice and Gjb6^–/–^ mice showed that Ca^2+^ waves failed to propagate in the Kölliker’s organ (Ortolano et al., 2008; Crispino et al., 2011), and consequently failed to acquire normal hearing (Sun et al., 2022). On the contrary, P2rx7 and Panx1, as alternative parts for ATP-triggered intercellular Ca^2+^ signaling, *P2rx7*^–/–^ (MGI:3606250) and *Panx*1^–/–^ (MGI:3606250) mice, showed that normal Ca^2+^ waves spread in Kölliker’s organ and normal hearing phenotype (Suadicani et al., 2006). Moreover, overexpression of Cx30 by transduction *in vivo* with BAAV (bovine adeno-associated virus) vectors encoding Cx30 *via* canalostomy at P4 not only restored calcium wave transmission in Gjb6^–/–^ mice but also partially and significantly improved hearing threshold around P30 (Crispino et al., 2011).

Although IHCs and OHCs do not express GJs, the Ribbon synapse of IHC retained immature morphology in Gjb2^loxP/loxP^; Sox10-Cre mice and Gjb6^–/–^ mice under transmission electron microscopy observation (Johnson et al., 2017). And patch clamp experiments also showed membrane currents and exocytosis capability of IHC retained at the prehearing stage (Johnson et al., 2017). The impaired synapse and nerve innervation of OHC also have been found in Cx26 deficiency mice (Johnson et al., 2017).

In conclusion, these results demonstrated that Cx-dependent ATP-triggered intercellular Ca^2+^ signaling pathway plays a key role in postnatal auditory development. Some scholars proposed the hypothesis that the disrupted Ca^2+^ signaling of developing cochlear epithelium prevents hearing acquisition in Cx26 deficiency mice (Ceriani et al., 2016; Johnson et al., 2017; Sun et al., 2022). However, the disrupted Ca^2+^ signaling hypothesis cannot explain the abnormal development of cochlear support cells, such as the failure of the tunnel of Corti to open and the failure of inner and outer column cells to differentiate and mature.

## Energy supply of the cochlear supporting cell *via* gap junction-mediated glucose transport pathway

The mammalian cochlear sensory epithelium is basically an avascular structure. With one exception, there is only one capillary, the spiral vessel, that traverses the sensory epithelium beneath the tunnel of Corti. The rest of the cochlear blood supply depends on two vascular networks, one serving the spiral limbus and another serving the spiral ligaments and SVs. The spiral ligament vessel, which is embedded in the fibrocytes forming part of the C-sys, crosses SVs and divides into many fine capillaries in the SVs.

Glucose transport is divided into two types, the family of glucose transporters (GLUT) that promote glucose diffusion along concentration gradient and sodium-dependent glucose transporters (SGLTs) that transport glucose against a concentration gradient (Mueckler and Thorens, 2013; Deng and Yan, 2016). Blood glucose is mainly transported along a concentration gradient, which does not require energy, but requires a carrier. In rats, the concentration of glucose in the perilymph of vestibular scala and media scala is only about 50% of that in blood plasma, while the glucose concentration in the endolymph is less than 10%. In 1983, Santos-Sacchi and Dallos discovered that GJs can help transport glucose and other metabolic substances to adjacent cells of the sensory epithelium (Santos-Sacchi and Dallos, 1983). Using a fluorescent glucose tracer (2-NBDG) which can monitor the ability of glucose uptake in living cells, it has been shown that glucose transport is through the intercellular GJs network system (Zou et al., 2005). Also in astrocytes, the network of GJs can transport energy and nutrients from the vascular zone to distant neurons in the avascular zone.

During cochlear development, both hair cells and supporting cells require a large amount of energy for differentiation and maturation. The insufficient energy supply will disturb normal development. Autophagy provides important energy for early development, and the deletion of autophagy-related molecules can be lethal to mouse embryos (He et al., 2017; Liu et al., 2021). Similarly, complete knockout of Cx26 mice has embryonic lethality, which is related to impaired transplacental glucose uptake (Bakirtzis et al., 2003). Interference with autophagy in early development also disrupts cochlear sensory epithelial development (Bu et al., 2022). In adult mice, the normal OHC electromotility activity also requires a large amount of energy, and the concertina movements of OHCs can reach 10,000 Hz frequency, and such a high frequency of cellular concertina movements must be accompanied by a large consumption of energy (Zhu et al., 2013). However, OHC electromotility does not depend on ATP but probably on the constant uptake of glucose from the cortilymph by the glucose transporters in the lateral walls of OHCs. Glucose of cortilymph may come from the hemichannel secretion of supporting cells.

Mutations in Cx26 reduce the coupling of CJs, which limits the transport of nutrients, especially glucose from distal vessels to avascular sensory epithelium. The glucose transport pathway mediated by GJs is critical for the differentiation and maturation of supporting cells, especially the inner and outer column cells of the tunnel of Corti during early development (Xie et al., 2019). Limited energy supply may hinder the formation of microtubules of inner and outer column cells, leading to failure to open the tunnel of Corti, which can explain the developmental disorder in congenitally profound deafness model mice (Lin et al., 2013; Xie et al., 2019). The lack of nutrients such as glucose affects ATP production, leading to reactive oxygen species (ROS) overload and cell apoptosis (Wang et al., 2016; Fetoni et al., 2018). This provides a mechanism to explain the massive loss of OHCs due to their high elevated levels of mitochondrial metabolism, making them more susceptible to intracellular ATP deprivation (Wang et al., 2022). Considering that OHCs in the high-frequency region require more energy, it could not only explain that Cx26 defect model mice with delayed progressive hearing loss that usually start to hearing impairment at the high frequencies but also explain that Cx26-related delayed hearing loss has noise susceptibility and age-related characteristics (Fetoni et al., 2018; Lin et al., 2019).

## Summary and outlook

Cx26 plays a critical role for hearing acquisition and maintenance. Cx26 mutations can induce congenital deafness and late-onset hearing loss. Cx26 is responsible for intercellular substance transfer and signal communication. GJ channels and hemichannels generally allow the passage of potassium, ATP-calcium signaling, and glucose. Potassium recycling is critical for maintaining EP and OHC electromotility. Therefore, Cx26 mutation can disrupt potassium recycling in cochlea lymph, leading to EP reduction and active cochlear amplification impairment. ATP-triggered intercellular Ca2+ signaling is critical for cochlear development. Cx26 mutation can lead to cochlear IHC development disorder. Cochlear development for hearing acquisition and OHC electromotility for hearing maintenance require sufficient energy supply, which depends on the cochlear supporting cell by GJ-mediated glucose transport pathway. Thus, energy deprivation at different periods due to Cx26 deficiency can cause cochlear non-sensory and sensory epithelial cell development arrest or OHC electromotility impairment with aging or noise.

Cx26 function has been studied for decades, most of which focus on downstream function changes. Nowadays, there are some advances in the treatment of *GJB2* mutation-related deafness. Yu et al. (2014) and Iizuka et al. (2015) used virally mediated gene therapy to restore Cx26 expression in a mouse model of Gjb2 deletion and improved the auditory responses or development of the cochlear structure. Xu et al. (2022) found that systemic administration of dexamethasone could prevent OHCs loss and improve auditory responses at some frequencies. Monoclonal antibodies developed in the last three decades have become the most important class of therapeutic biologicals (Buratto et al., 2021). Ziraldo et al. (2019) found that a human-derived monoclonal antibody named abEC1.1 can selectively modulate hemichannel function and efficiently inhibit hyperactive mutant Cx26 hemichannels implicated in autosomal dominant non-syndromic hearing impairment accompanied by keratitis and hystrix-like ichthyosis-deafness (KID/HID) syndrome (Xu et al., 2017). So far, there is no drug to prevent or treat Cx26 mutation-related hearing loss. Post-translational modifications of proteins can regulate the Cx26 protein life cycle and/or channel selective permeability by the covalent addition of functional groups or proteins, changing the hydrophilicity and spatial structure of Cx26. The upstream molecular regulation mechanism of Cx26 deserves further study to find more information for novel protective or therapeutic strategies to prevent or treat hereditary deafness caused by GJB2 mutation.

## Author contributions

PC: study conception and write the manuscript. WW and JZ: study conception and draft the manuscript and figures. JC, YL, and LS: study conception and screening. SH: quality control and write the manuscript. JY: study conception, quality control, and write the manuscript. All authors contributed to the article and approved the submitted version.
